# Cervical cancer knowledge, awareness and related health behaviours amongst women of reproductive age in Kiambu County, Kenya: a cross-sectional study

**DOI:** 10.1080/21642850.2022.2136184

**Published:** 2022-10-21

**Authors:** Eliphas Gitonga, Rosebella Iseme, Redempta Mutisya, Maurice Kodhiambo

**Affiliations:** a Department of Population and Reproductive Health, School of Public Health and Applied Human Sciences, Kenyatta University, Nairobi, Kenya; b, Department of Population Health, Medical College, Aga Khan University, Nairobi, Kenya; c Department of Pharmacognosy, Pharmaceutical Chemistry and Pharmaceutics, School of Pharmacy, Kenyatta University, Nairobi, Kenya

**Keywords:** Cervical cancer screening, HPV, knowledge, attitudes, barriers

## Abstract

**Background:**

Data on cervical cancer knowledge, perceptions, screening practices and other relevant health behaviours among women in rural Kenya is limited. Yet understanding this information is a key first step in developing evidence-based interventions aimed at addressing the low uptake of screening services and heavy cervical cancer disease burden within Kenya. Consequently, our study sought to assess cervical cancer knowledge, attitude, and practice amongst women of reproductive age within Kiambu County, known for a high cervical cancer disease burden.

**Methods:**

This was an analytical cross-sectional study undertaken in April 2022. Data was collected using interviewer-administered questionnaires from 472 females randomly selected from within the community. Data analysis included descriptive statistics (mean values, standard deviations, and frequencies) and logistic regression, using STATA version 13.

**Results:**

More than 80% of respondents were aware of cervical cancer though only 54% answered at least half of the knowledge questions correctly. Knowledge of HPV was particularly low, likely because 55% of the study sample stated they had never heard of HPV. Though 89% of study participants deemed cervical cancer preventable, more than 60% had an unfavourable attitude towards cervical cancer screening, deeming the process expensive, painful, and embarrassing. In line with the latter observation, only 20% of our sample had ever been screened for cervical cancer and less than half of this group had undergone regular screening. Notably, knowing a place where cervical cancer screening services are provided had the largest increase in odds of being screened (3.94; 95% CI: 1.08–14.37). Fear of tests and outcomes was also noted to be a prime concern amongst our study participants.

**Conclusion:**

A clear message from this study is the need to ensure community members are aware of where to access screening services and strategies are implemented to address prevalent fears and negative perceptions.

Abbreviations: CHV: Community Health Volunteers; HPV: Human papillomavirus; HIV/AIDS: Human immunodeficiency virus/Acquired immune deficiency syndrome; LEEP: Loop Electrosurgical Excision Procedure; LMICs: Low- and Middle-Income Countries; NCI: National Cancer Institute; NACOSTI: National Commission for Science, Technology, and Innovation; VIA: Visual inspection with acetic acid; VILLI: Visual inspection with Lugol’s iodine; WHO: World Health Organisation

## Background

Cervical cancer is the third most common cancer among women globally, with 569,847 new cases and 311,365 deaths reported in 2018 (Bray et al., 2018). Women in low and middle-income countries (LMICs) bear a disproportionately heavy burden of cervical cancer as it accounts for more than 80% of new cases and 90% of all cervical cancer deaths (Sung et al., 2020). In Kenya, cervical cancer is the second leading type of female cancer (after breast cancer) and the most common type in women aged 15–44 years (ICO/IARC Centre on HPV and Cancer, 2021).

Cervical cancer is mostly caused by a persistent infection with any of twelve specific carcinogenic types of human papillomavirus (HPV) that are sexually transmitted by both sexes (Chan et al., 2019; Wild et al., 2020). Two common high-risk HPV types (16 and 18) are responsible for approximately 70% of cervical cancers worldwide. Whilst most HPV infections are transitory, in some women they persist, especially if they are HIV positive (Chan et al., 2019; Wild et al., 2020). HPV-16/18 infection is reported to occur in an estimated 9.1% of women and cause 63.1% of invasive cervical cancers in Kenya (ICO/IARC Information Centre on HPV and Cancer, 2021).

The key strategies to combat the incidence and mortality of cervical cancer within the country in accordance with the Kenya National Cancer Control Strategy (NCCS) 2017–2022 include HPV vaccines as primary prevention, and cervical cancer screening to identify early changes in cells as secondary prevention with subsequent treatment (Chan et al., 2019; Ministry of Health, Kenya, 2017). Early detection can result in better treatment outcomes, less morbidity, and even lower costs of treatment. Cervical cancer screening has been shown to reduce cervical cancer incidence as well as result in a 41–92% mortality reduction (Jansen et al., 2020; Landy et al., 2016). The National Cancer Screening Guidelines in Kenya identify women aged 25–49 years as the target population for screening (Ministry of Health, Kenya, 2018). The guidelines recommend HPV testing as the primary screening method; visual inspection with acetic acid (VIA) alone or combined with visual inspection with Lugol’s iodine (VILI) where facilities for HPV testing are not yet available, or pap smear in some specified circumstances (Ministry of Health, Kenya, 2018). Ideally, a same day ‘screen and treat’ approach with cryotherapy and/or Loop Electrosurgical Excision Procedure (LEEP) is recommended (Ministry of Health, Kenya, 2018).

The high burden of cervical cancer in LMICs is linked to a lack of utilisation of screening, with the proportion screened ranging between 3% and 14% (Bruni et al., 2021; Huchko et al., 2020). The latter is true in Kenya, where despite the existence of preventive programmes, cervical cancer screening rates have remained relatively low with screening facilities underutilised (Ministry of Public Health and Sanitation & Ministry of Medical Services, 2012; Vermandere et al., 2016). Following, the introduction of cervical cancer screening programmes in 2013 and HPV vaccination in 2019 in Kenya, only 13.8% of women in the reproductive age participate in screening (this number drops below 11% in rural areas) and nearly 50% of women still present with late-stage disease (Donkor et al., 2015; Kenya Bureau of Statistics, 2016; Ministry of Health, Kenya, 2019). If current trends are left unchecked, 98% of cervical cancer mortalities will be from the developing nations by the year 2030 (Lemp et al., 2020). According to the National Cancer Institute (NCI) of Kenya, Kiambu County is amongst the top 10 counties with the highest prevalence of cervical cancer (Awich, 2019). In fact, a recent study assessing the trend in cancer cases diagnosed at Kiambu and Gatundu Level 5 hospitals in Kiambu County between 2013 and 2017, found cervical cancer to be the leading cancer across both facilities making up 33.3% of cases (Warui et al., 2021). Furthermore, cervical cancer screening utilisation is noted to be low, despite health centres in Kiambu County having integrated screening services for cervical cancer (Kanyina et al., 2017; Wanyoro & Kabiru, 2017).

Understanding the level of knowledge and attitude of women on issues related to cervical cancer is a key first step in the development of evidence-based interventions aimed at addressing the low uptake of screening services and heavy cervical cancer disease burden within Kenya. Notably, recent studies assessing cervical cancer knowledge and attitude in Kenya have focused on women aged 25–49 years (most likely due to existing screening guidelines) with limited population-based studies focussed on pre-screening age groups (15–24 years) (Mugai et al., 2020; Sakwa et al., 2021). Yet, young women aged 15–24 years, represent an ideal target group for behavioural interventions to prevent cervical cancer in Kenya because of their developmental stage and the fact that most are sexually active (Ngune et al., 2020). Understanding all the contextual issues that may contribute to effective prevention of this disease including lack of awareness and knowledge as well as risky health behaviours amongst this target population can give insights into various ways policymakers and researchers could address the burden of cervical cancer amongst our population. Our study, therefore, sought to assess the level of knowledge, attitude and related health behaviours of women of reproductive age (15–49 years) in Kiambu County in relation to cervical cancer.

## Materials and methods

### Study design and period

An analytical cross-sectional study design was utilised (data collection in the month of April 2022).

### Study area

Kiambu County is one of Kenya’s 47 counties. It is located in the central region and neighbours Nairobi. It is the second-most populous county after Nairobi City County with a total population of 2,417,735 (51% of which are female; 26.9% are females in the reproductive age). The county has a high population growth rate (at 2.81%). It has 12 sub-counties that are further divided into 60 wards.

Kiambu sub-county was selected as the study area as it is more metropolitan comprising of a core urban area surrounded by rural settings. It has four wards (Ndumberi, Riabai, Township and Ting’anga). The rural areas constitute coffee plantations where women work while the urban area (Township) mainly borders Nairobi. A map of the administrative units in Kiambu County is presented in Appendix 1.

### Sample size

The sample size was determined using Fisher's formula for sample size calculation at a 95% level of confidence. We applied the assumption that the fraction of the population with preferred characteristics was unidentified within the selected study site, in turn using 50% as the fraction of people with preferred prevention practices. After accounting for non-response and data loss (incomplete data) (25%), a minimum sample size of 482 study participants was required for this study (refer to Appendix 2 for sample size calculation).

### Sampling technique

Random sampling was utilised to select specific households from all four wards where a single woman from each selected household was interviewed. In households with more than one eligible woman, random sampling was used to select one female respondent and where a household did not have an eligible participant, the next household was selected.

### Data collection tools and procedures

Data was collected using an interviewer-administered semi-structured questionnaire by trained research assistants (data captured using Kobo Collect software and exported to STATA).

### Data quality control

During the data collection exercise, the principal investigator undertook real-time checking of the data in the web system for any errors in data entry and transmission with prompt feedback to the research assistants in the field whenever any issues were noted. The form was loaded in the software after pre-testing it. Any changes in the form were communicated to the data collection team simultaneously to ensure reliability of the data.

### Data analysis

Quantitative data analysis was performed using STATA version 13 (StataCorp, 2013). Analyses of baseline characteristics treated scores as either continuous or categorical measures. Descriptive statistics such as means, standard deviations, frequencies, and percentages (where applicable) were used to describe the distribution of knowledge, attitude and practice characteristics amongst our study sample. Univariate and multivariable logistic regression analyses were used to assess the relationship between sociodemographic characteristics, participant awareness, level of knowledge and attitude towards cervical cancer-related factors and cervical cancer screening behaviour. Only variables that were observed to have a statistically significant association in the univariate analyses were added to the multivariable model. Analyses were undertaken on those with complete data, i.e. those who provided responses to all questions posed. All estimates are presented with 95% confidence intervals and (2-sided) *p* values. Significance was set at *p* < .05.

### Ethical considerations

The proposal received ethical clearance from Kenyatta University Ethics and Research Committee (Approval number: PKU/2333/11472). All necessary approvals were sought including that of the National Commission for Science, Technology, and Innovation (NACOSTI). Informed consent from all study participants was obtained (refer to Appendices 3, 4 and 5). Additional measures taken to protect respondent’s privacy and confidentiality, included the collection of de-identified data (refer to Appendix 6), encryption and password protection of all electronic data files and maintenance of paper-based data files (consent forms) in locked filing cabinets in locked rooms under the supervision of the principal investigator.

## Results

### Demographic characteristics

A total of 472 women agreed to participate in the study, i.e. Riabai (*n* = 136), Ndumberi (*n* = 98), Township (*n* = 98) and Ting’ang’a (*n* = 140), representing a 98% response rate. Table 1 presents a summary of study participant demographic data.

**Table 1.** Demographics characteristics (*n* = 472).

Demographic characteristic	Mean (±SD); *n* (%)
Age	31 years (±8.9 years)
	Below 25 years – 132 (27.97%)
	25 years and above – 340 (72.03%)
Religion	**Protestant – 326 (69.07%)**
	Catholic – 132 (27.97%)
	Muslim – 1 (0.21%)
	Other – 13 (2.75%)
Occupation	**Unemployed – 203 (43.01%)**
	Self-employed – 168 (35.59%)
	Employed – 45 (9.53%)
	Casual – 56 (11.86%)
Level of education	No formal education – 14 (2.97%)
	Primary – 176 (37.29%)
	**Secondary – 192 (40.68%)**
	Tertiary – 90 (19.07%)
Marital status	Single (never married) – 151 (31.99%)
	**Married – 273 (57.84%)**
	Separated – 39 (8.26%)
	Widowed – 5 (1.06%)
	Divorced – 4 (0.85%)
Sexual partner	No – 110 (23.30%)
	**Yes – 311 (65.89%)**
	No response – 51 (10.80%)
Ever had a family member diagnosed with cervical cancer	Yes – 46 (10%)
Ever had a friend diagnosed with cervical cancer	Yes – 88 (18.64%)
Source of payment for health services	Employer health expenditure – 4 (0.85%)
	Private health expenditure – 6 (1.27%)
	**Out of pocket (self or family member) – 341 (72.25%)**
	NHIF – 121 (28.34%)

More than half of our sample (72.03%) was aged above 25 years old and had completed secondary education (40.68%). Similarly, more than half of our sample was married (57.84%) and majority were unemployed (43.01%). An even larger proportion of our sample acknowledged having a sexual partner (65.89%), whilst only 28.39% knew someone (family member or friend) that had been diagnosed with cervical cancer. Notably, the predominant source of payment for health services was out of pocket (72.25%).

### Awareness and knowledge

Awareness of cervical cancer was high amongst our study sample. Though majority of study participants stated they had heard of cervical cancer (86%) and cervical cancer screening (70.97%), only 62.92% of respondents were aware of a place that offers cervical cancer screening services. The most commonly identified place was a public hospital (*n* = 173; 58.25%)

Awareness of HPV and associated factors was observed to be much lower, with only 44.70% having heard of HPV and even fewer of HPV testing (27.33%) and vaccination (40.04%). Study participants’ responses to various awareness statements are presented in Appendix 7.

Table 2 shows the percentage of women who answered 10 questions assessing participant knowledge of cervical cancer-related factors correctly. Out of a possible 10 marks (1 mark for each question), the mean score was 4.5 (±3), with a minimum score of 0 (*n* = 75; 15.89%) and a maximum of 10 (*n* = 2; 0.42%), whilst 255 (54.02%) study participants scored at least 5/10. Study participants were least knowledgeable about the relationship between multiparity and risk of cervical cancer, and genetics and risk of cervical cancer. Whilst majority of study participants appeared knowledgeable regarding the risk of cervical cancer associated with having multiple sexual partners or a partner with multiple sexual partners.

**Table 2.** Summary of participant knowledge of cervical cancer-related factors (*n* = 472).

Knowledge characteristics	*n* (%)
You are more likely to get cervical cancer if your family member has it	127 (26.91%)
If a woman has an abnormal cervical cancer screening result, she will definitely have cervical cancer	154 (32.63%)
HPV infection increases the risk/probability of developing cervical cancer	191 (40.47%)
Early initiation of sexual intercourse increases the risk of getting cervical cancer	250 (52.97%)
Having multiple sexual partners increases the risk of getting cervical cancer	296 (62.71%)
Having a sexual partner with multiple sexual partners increases the risk of getting cervical cancer?	285 (60.38%)
Co-infection with other sexually transmitted infections increases the risk of getting cervical cancer	264 (55.93%)
Having many children/multiparity increases the risk of getting cervical cancer	79 (16.74%)
Immunosuppression due to HIV/AIDS infection increases the risk of getting cervical cancer	23 (70.21%)
Tobacco use increases the risk of getting cervical cancer	252 (53.39%)

Overall, the level of knowledge on HPV and related factors was low, most likely affected by the fact majority of the sample (*n* = 216; 55.30%) stated they had never heard of HPV. In fact, the study results show even though study participants were largely aware of the role of risky sexual behaviour in cervical cancer development, they were largely unaware that HPV was the causative factor in that relationship. Notably, the largest knowledge gap lay in the fact majority of our sample (*n* = 466; 98.73%) believed that HPV infection always had visible signs and symptoms. Appendix 8 shows the percentage of women who answered the 17 questions assessing knowledge of HPV-related factors correctly. Notably, out of a possible 17 marks (1 mark for each question), the mean score was 3.3 (±4.1), with a minimum score of 0 and a maximum of 15.

### Attitude

Table 3 shows the percentage of women who agreed or strongly agreed with 8 items assessing participant attitude towards cervical cancer-related factors. Majority of respondents had a favourable attitude towards cervical cancer with more than three-quarter of study participants (89.39%) agreeing with the idea that cervical cancer can be prevented. Alternatively, majority of respondents had an unfavourable attitude towards cervical cancer screening, with majority agreeing that cervical cancer screening is expensive (64.20%), painful (66.94%) and embarrassing (64.83%). Despite this majority (84.11%) generally agreed that cervical cancer screening helps in the prevention of cervical cancer.

**Table 3.** Summary of participant attitude towards cervical cancer-related factors (*n* = 472).

Attitude characteristics	*n* (%)
Cervical cancer is a deadly disease	429 (90.89%)
Cervical cancer can be prevented	421 (89.39%)
Any woman who has ever had sexual intercourse should be screened for cervical cancer	418 (88.55%)
Screening helps in prevention of cervical cancer	397 (84.11%)
Screening for cervical cancer is expensive	303 (64.20%)
Cervical cancer screening can be a painful test	316 (66.94%)
Cervical cancer screening is embarrassing	306 (64.83%)
Cervical cancer screening takes a lot of time	235 (49.79%)

### Health behaviours

Amongst the 340 women aged 25 years and above, only 20.29% (*n* = 69) had ever undergone screening for cervical cancer. Pap smear (*n* = 30) and Via Vili (*n* = 23) were the common strategies used. Notably, majority (*n* = 68; 98.55%) of the women who had undergone screening stated they would recommend the type of screening they received to other women. Furthermore, amongst those who have ever undergone screening for cervical cancer (*n* = 69), 15 (21.74%) reported undergoing screening annually, 20 (28.99%) reported undergoing screening every five years and 17 (24.64%) stated they underwent screening once in a while. 17 (24.64%) either did not know or could not remember how often they undergo cervical cancer screening.

Regarding condom use, 45.34% (*n* = 214) of study participants stated that they ‘never use a condom’. Notably, majority of women who reported ‘non-use of condoms’ were married (86.45%; *n* = 185); whilst 22 (10.28%) were single (never married), 5 (2.34%) were separated; 1 (0.47%) was divorced and 1 (0.47%) was a widow. Similarly, amongst those who stated they ‘sometimes used a condom’, 45 (52.94%) were married, 33 (38.82%) single, and 7 (8.23%) were separated. Whilst, amongst the 24 women that reported ‘always using a condom’, 6 (25%) were married, 13 (54.17%) single and 5 (20.83%) separated.

A total of 126 (26.69%) women reported having experienced a reproductive tract infection in the last 5 years. Amongst these 112 (88.89%) had sought treatment for the infection. Data on study participants’ health behaviours is presented in Table 4.

**Table 4.** Summary of participant health behaviours.

Health behaviours	*n* (%)
Cervical cancer screening (*n* = 340)	**No – 271 (79.71%)**
	Yes – 69 (20.29%)
Condom use (*n* = 472)	**Never – 214 (%)**
	Sometimes – 85 (%)
	Always – 24 (%)
	No response – 149 (%)
Treatment for reproductive tract infections occurring in the last 5 years (*n* = 126)	No – 9 (7.14%)
	**Yes – 112 (88.89%)**
	No response – 5 (3.97%)
Intent to test for HPV (*n* = 472)	Definitely yes – 182 (38.56%)
	**Maybe – 214 (45.34%)**
	No – 76 (16.10%)

Health behaviours were also observed to cluster. In this regard, majority of those who didn’t use condoms also had never been screened for cervical cancer (i.e. 182/214). In fact, only 15.76% (i.e. 49/311 study participants) of respondents who stated they had a sexual partner also stated they had ever undergone cervical cancer screening.

Interestingly, only 38.56% (*n* = 182) of study respondents answered ‘definitely yes’, when asked whether they would be willing to get tested if HPV testing was made available to them.

Univariate analysis showed a statistically significant association between screening behaviour and age; marital status; occupation; awareness of cervical cancer, cervical cancer screening and a place where cervical cancer screening services are provided as well as awareness of HPV, HPV testing and vaccination. Also, knowledge of cervical cancer-related factors, knowledge of HPV-related factors and attitude towards cervical cancer-related factors were significantly associated with having previously undergone cervical cancer screening (refer to Appendix 9). In the multivariable logistic model only age; awareness of cervical cancer screening, HPV testing and vaccination; knowing a place where cervical cancer screening services are provided as well as knowledge of and attitude towards cervical cancer-related factors remained statistically significantly associated with screening behaviour. The latter results are summarised in Table 5.

**Table 5.** Multivariable analysis assessing the relationship between study participant characteristics and screening behaviour (*n* = 340).

Variables	Crude OR (95% CI)	*p* Value	Adjusted OR (95% CI)	*p* Value
*Age*	1.09 (1.06–1.12)	.000	1.05 (1.01–1.10)	.017*
*Occupation*				
Unemployed	1		1	
Employed	1.87 (0.77–4.55)	.166	1.49 (0.48–4.63)	.495
Self-employed	2.53 (1.42–4.52)	.002	1.28 (0.58–2.85)	.542
Casual	1.44 (0.60–3.46)	.410	1.06 (0.36–3.13)	.911
*Marital status – n (%)*				
Single (never married)	1		1	
Married	1.93 (1.02–3.65)	.043	0.67 (0.30–1.50)	.328
Separated/divorced/widowed	4.89 (2.17–11.04)	.000	1.76 (0.58–5.31)	.316
*Ever heard of cervical cancer*	6.76 (1.62–28.25)	.009	0.53 (0.07–3.87)	.534
*Ever heard of cervical cancer screening*	26.24 (3.60–191.48)	.001	9.33 (1.05–82.82)	.045*
*Know a place for cervical cancer screening*	12.65 (3.90–41.05)	.000	3.94 (1.08–14.37)	.038*
*Ever heard of HPV*	4.25 (2.45–7.36)	.000	0.63 (0.18–2.15)	.457
*Ever heard of HPV testing*	4.86 (2.90–8.14)	.000	2.49 (1.03–6.0)	.043*
*Ever heard of HPV vaccination*	5.40 (3.11–9.38)	.000	2.88 (1.24–6.70)	.014*
*Knowledge of cervical cancer-related factors*	1.33 (1.20–1.47)	.000	1.21 (1.05–1.40)	.010*
*Knowledge of HPV-related factors*	1.18 (1.12–1.25)	.000	0.93 (0.82–1.06)	.292
*Attitude towards cervical cancer-related factors*	1.81 (1.50–2.18)	.000	1.54 (1.23–1.94)	.000*

### Partner support and barriers to accessing health services

Only 59 (12.5%) women reported ever receiving partner support towards accessing cervical cancer screening. The nature of support received was varied and included the provision of moral support, financial support, the male partners providing information or company to their wives during hospital visits. Moral support was the most predominant form of support received by the women from their partners. Moral support includes approval for the testing, encouragement to have the test done, and assurance of support with whichever outcome of the test (whether positive or negative). Appendix 10 illustrates the distribution of the different types of support received by the 59 women.

In addition, Appendix 11 outlines the range of barriers identified by our study participants as factors impeding their access to cervical cancer screening. These barriers can be broadly divided into personal and structural impediments. Amongst personal impediments, fear of the tests and outcomes was noted to be the single most prevalent barrier, alongside lack of information. Surprisingly, structural factors such as the costs of tests did not factor as prominently as personal impediments. In fact, only two study participants selected ‘shortage of personnel’ as a barrier. A range of other barriers were listed by study participants. These included, fear the healthcare worker may disclose their results, their own personal beliefs, and their religion.

The low event rate alongside the various responses to partner support and barriers that would have likely biased out results prevented us from undertaking regression analysis.

## Discussion

This study sought to describe the level of knowledge, attitude and health behaviours related to cervical cancer within Kiambu County. Cervical cancer is the most common cancer observed amongst patients presenting in facilities within the County (Warui et al., 2021). Kiambu County is also amongst the top 10 counties with the highest prevalence of cervical cancer within Kenya (Waitara, Kerich, Kihoro, & Korir, 2021). According to a recent study, 70–80% of people in the County visit the screening centres when the disease has significantly progressed making it challenging to contain or treat (Kanyina et al., 2017). Kiambu sub-county was chosen as the focus of this study as it is characterised by a predominantly rural morphology, making this population especially vulnerable, as coverage and utilisation of health services is often much lower than observed in their urban counterparts.

Our study findings revealed that only 20.29% of those targeted for screening have ever undergone cervical cancer screening, and almost half of the latter had done so irregularly. Most notably, only 38.77% (*n* = 183) of study participants showed a willingness to be screened for cervical cancer if the service was made available to them. Interestingly ‘age’ was the only sociodemographic factor independently associated with screening behaviour (AOR = 1.09; *p* = .000; 95% CI: 1.06–1.12). Other studies undertaken in LMICs also illustrate that ‘age’ is significantly associated with cervical cancer screening behaviour, with the mean age amongst those screened being higher than those not screened (Chang et al., 2017; Issa et al., 2021; George, 2021; Tekle et al., 2020). The latter may be explained by increased access to resources (such as decision-making power, finance, and information) amongst older women.

Regarding participant awareness, those who had heard of cervical cancer screening, those who knew a place where screening services were provided, as well as those who had heard of HPV testing and vaccination were more likely to have undergone cervical cancer screening. A high level of awareness of cervical cancer and related factors has been reported amongst adult women in KAP studies undertaken in Tharaka Nithi and Isiolo Counties within Kenya (80%), Southern India (82.9%) and Eastern Uganda (88.2%) (Gatumo et al., 2018; Ghosh et al., 2021; Mukama et al., 2017). In addition, familiarity with HPV vaccines has been shown to influence cervical cancer preventive behaviour in studies undertaken amongst Zambian women (Liu et al., 2012; Nyambe et al., 2019). The latter perhaps highlights the benefits of integrated screening and vaccination services often accompanied by health education (Rabil et al., 2022). Notably, though awareness is a vital health promotion tool, alone it is often unable to influence health practices (Arlinghaus & Johnston, 2017). Furthermore, a high level of awareness of cervical cancer and related factors has not been uniformly reported across all studies undertaken in similar populations (Mengesha et al., 2020). Interestingly, awareness of a place where screening services are provided had the largest increase in odds of being screened (AOR = 3.94; *p* = .038; 95% CI: 1.08–14.37).

Similarly, knowledge of cervical cancer-related factors was also significantly associated with screening behaviour (AOR = 1.21; *p* = .010; 95% CI: 1.05–1.40). The poor level of knowledge regarding the etiological role of viral infection in cervical cancer development and disease presentation noted amongst our study sample has been previously reported (Dozie et al., 2021; Ghosh et al., 2021; Phaiphichit et al., 2022). In fact, a study undertaken in Nigeria reported that majority of the sample strongly agreed that if they did not feel discomfort or pain there was no need for cervical cancer screening (Dozie et al., 2021). Similarly in our sample ‘HPV infection always has visible signs and symptoms’ was noted to be a large knowledge gap that may explain the low levels of utilisation observed. In fact, the absence of clinical signs and symptoms has been reported as a common reason for not being screened in a study undertaken amongst women aged 25–60 years in the Lao People’s Democratic Republic (Phaiphichit et al., 2022).

Cervical cancer is a preventable and curable disease if detected early and managed effectively (WHO, 2022). To attain the goal, set out by the World Health Organisation (WHO) to reach and maintain an incidence rate of below 4 per 100,000, countries require strategic action. This strategic action is proposed to rest on three main pillars (WHO, 2020); first, prevention through HPV vaccination, second, screening and treatment of precancerous and third, for those who are identified with invasive cancer, timely care and treatment saves lives, while palliative care can greatly reduce pain and suffering. All three pillars must be implemented collectively and at scale to achieve the goal of elimination. Notably, a key step in ensuring these strategies are effective in eliminating cervical cancer cases is the need to improve public knowledge, awareness and attitude regarding cancer; alongside investing in strengthening and equipping health services, training health workers so they can conduct accurate and timely diagnostics, as well as ensuring people living with cancer can access safe and effective treatment, including pain relief, without incurring prohibitive personal or financial hardship. To date majority of the emphasis on these efforts has focused on addressing the latter two structural factors. Notably, our results indicated that structural factors did not feature as prominently as personal impediments as perceived barriers affecting study participants ability to access cervical cancer screening services. This would explain the fact that despite the provision of free screening programmes, some women still fail to take advantage of these opportunities in turn highlighting the need for greater emphasis on educational interventions (Ng’ang’a et al., 2018). Notably, previous research has highlighted the fact that the effectiveness of health education and subsequent improved knowledge on behaviour change depends on people’s beliefs on a range of factors including the disease and their self-efficacy (Zimmerman, 2000). Beliefs provide the cognitive basis of an attitude. Notably, attitude towards cervical cancer-related factors was also statistically significantly associated with screening behaviour amongst our sample (AOR = 1.54; *p* = .000; 95% CI: 1.23–1.94). The latter association was also noted in a study undertaken amongst 25 low-, middle-income and emerging-economy countries, however, the samples observed in the latter populations were much younger (mean age = 20.9; SD = 2.0) (Pengpid & Peltzer, 2014).

Current government actions, such as allocating financial and human resources to the National Cancer Institute-Kenya and the National Cancer Control Program, are indicative of a genuine intention to improve the delivery of cancer services in the country (Makau-Barasa et al., 2020). However, it is clear, our study population may benefit from health communication efforts aimed at raising awareness regarding the fact that prognosis is significantly improved through early detection. In this regard, the country’s existing community health strategy (Ministry of Health, Kenya: Division of Community Health Services, 2020) recognised as the country’s first level of healthcare, may offer an ideal opportunity to deliver this health-promotive activity, given the acknowledged shortage of personnel in the country’s cancer sector (Makau-Barasa et al., 2020). Through this strategy, Community Health Volunteers (CHVs) operate within the community making home visits to deliver health promotion messages and link members of the community to health facilities. Notably, areas within the country that have an active community health programme have already demonstrated improvements in antenatal care visits, testing and treatment for diseases like HIV and malaria, and child immunizations (Huchko et al., 2018).

## Limitations

The results of this study should be interpreted in light of certain limitations. First, as a cross-sectional survey, our quantitative data may have been limited by selection bias and our findings are purely hypothesis generating. Furthermore, as we were interested in seeing if any trends emerged across groups, we did not collapse groups together, which sometimes resulted in low event rates and wide confidence intervals. Furthermore, no qualitative data was collected to enable a deeper understanding of the various barriers that were raised by study participants. Also, studies collecting information on sexual lifestyle are often deemed sensitive. Study participants are not always willing to provide information on these issues, whilst social desirability bias may influence the validity of responses provided by those who choose to answer sensitive questions. Lastly, our data collection took place in only one sub-county in Kiambu. The sample may therefore not be representative of the whole County.

## Conclusion

Early cervical cancer screening and treatment is an effective preventive method, however, uptake of this service continues to be poor. Determining the level of awareness, knowledge and attitudes related to cervical cancer are the first steps in establishing locally relevant interventions aimed at reducing the burden of this disease amongst our population. A clear message from this study is the need for strategies aimed at raising awareness and knowledge particularly on where screening services may be accessed as well as the benefits of cancer screening to address prevalent fears and negative perceptions amongst members of the community. This is key if public health activities such as the provision of free screening services are to bear fruit in terms of reducing disease burden. Future research should be encouraged to evaluate community-based interventions that have the potential for greater impact.

## Supplementary Material

Supplemental Material
