# Supplementary material for: Cervical cancer knowledge, awareness and related health behaviours amongst women of reproductive age in Kiambu County, Kenya: a cross-sectional study
Source: Health Psychol Behav Med. 2022 Oct 21;10(1):1056–70. doi: 10.1080/21642850.2022.2136184 (PMC9590427; doi:10.1080/21642850.2022.2136184)
Supplement: Supplemental Material [file RHPB_A_2136184_SM8120.docx]

## Appendices

## Appendix 1: Map of Kiambu County showing the administrative units


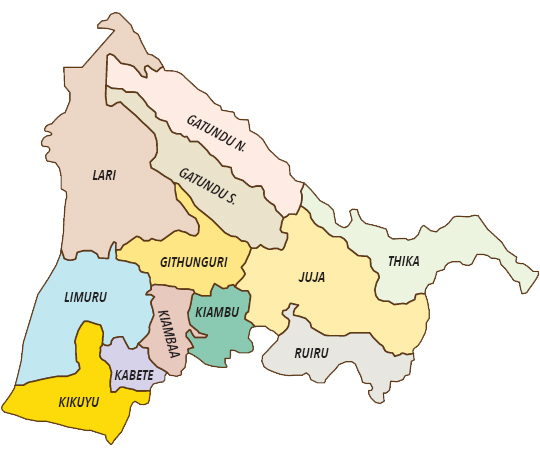


## Appendix 2: Sample size calculation

- n = (Z /∆)² × p(1-p)
  - p = 0.50
  - Z = 1.96
  - ∆ = 0.05
- n = (1.96 /0.05)² * 0.50(1-0.50)
- n = (39.2) ² × 0.25
- n = 1,536.64 × 0.25
- n = 384.16 = 385
- 385 × 1.25 = 481.25 = 482

## Appendix 3: Adult Participant Consent Form

**Project Title:** _____A Multi-modal Intervention to Accelerate Utilization of Human Papilloma Virus Screening for Cervical Cancer in Kiambu County, Kenya _______________________________________________

**Investigator(s):** ____________________

We are doing a research study to help us better understand the utilization of HPV screening amongst women of reproductive age in Kiambu County.

Permission has been granted to undertake this study by ____________________.

If you decide that you want to be part of this study, you will be asked some questions and the information you provide will be recorded in a questionnaire.

There are some things about this study you should know. Some of the questions you will be asked are of an intimate nature and may be embarrassing or make you uncomfortable. If this happens, you may refuse to answer these questions if you so choose. You may also stop the interview at any time. The interview will take approximately half an hour of your time.

If you participate in this study you will help us to learn how to provide effective screening services that can improve health of women.

There are no rewards or any payment to you if you participate.

To protect your privacy, your name will not be recorded on the questionnaire. The questionnaires collected will also be kept confidential and only shared with the study team. When we are finished with this study we will write a report about what was learned. This report will not include your name or that you were in the study.

You do not have to be in this study if you do not want to be. If you decide to stop after we begin, that’s okay too.

If you have questions about the study, you can reach the Principal Investigator, ____________________.

However, if you have questions about your rights as a study participant: You may contact the Ethical Review Committee Secretariat on ____________________

If you decide you want to be in this study, please sign below.

Signing below is an indication; that the nature of the study has been fully explained to you and you have been given a chance to ask questions and your questions have been answered to your satisfaction; that your participation in this study is entirely voluntary; that you understand that your records will be kept confidential and that you can leave the study at any time.; and that the information provided has enabled you to make a fully informed decision concerning your involvement in the study.

A copy of this consent form will be provided to you.

_____________________________________

Name of Participant

_____________________________________

Signature or Thumbprint

______________________________

Date

**Investigators Statement**

I, the undersigned, have explained to the volunteer in a language she/he understands the procedures to be followed in the study and the risks and benefits involved.

_____________________________________

Name of Interviewer (Research Assistant)

_____________________________________

Signature

______________________________

Date

(This signed form affirms subject eligibility for the study and that informed consent has been obtained.)

## Appendix 4: Parental Consent Form

**Project Title:** _____A Multi-modal Intervention to Accelerate Utilization of Human Papilloma Virus Screening for Cervical Cancer in Kiambu County, Kenya _______________________________________________

**Investigator(s):** ____________________

We are doing a research study to help us better understand the utilization of HPV screening amongst women of reproductive age in Kiambu County.

Permission has been granted to undertake this study by ____________________

If you permit your daughter to be part of this study, she will be asked some questions and the information she provides will be recorded in a questionnaire.

There are some things about this study you should know. Some of the questions that will be asked are of an intimate nature and may be embarrassing or make her uncomfortable. If this happens, she can refuse to answer these questions if she so chooses. She may also stop the interview at any time. The interview will take approximately half an hour of her time.

If she participates in this study her information will help us to learn how to provide effective screening services that can improve health of all women young and old.

There are no rewards or any payment to her or yourself if she participates.

To protect her privacy, her name will not be recorded on the questionnaire. The questionnaires collected will also be kept confidential and only shared with the study team. When we are finished with this study we will write a report about what was learned. This report will not include her name or that they were in the study.

She does not have to be in this study if she does not want to be. If she decide to stop after we begin, that’s okay too.

If you have questions about the study, you can reach the Principal Investigator, ____________________.

However, if you have questions about your child’s rights as a study participant: You may contact the Ethical Review Committee Secretariat on ____________________.

If you decide you permit your child to be in this study, please sign below.

Signing below is an indication; that the nature of the study has been fully explained to you and you have been given a chance to ask questions and your questions have been answered to your satisfaction; that your decision to allow your child to participate in this study is entirely voluntary; that you understand that your child’s records will be kept confidential and that she can leave the study at any time.; and that the information provided has enabled you to make a fully informed decision concerning her involvement in the study.

A copy of this consent form will be provided to you.

_____________________________________

Name of Participant

_____________________________________

Signature or Thumbprint

______________________________

Date

**Investigators Statement**

I, the undersigned, have explained to the volunteer in a language she/he understands the procedures to be followed in the study and the risks and benefits involved.

_____________________________________

Name of Interviewer (Research Assistant)

_____________________________________

Signature

______________________________

Date

(This signed form affirms subject eligibility for the study and that informed consent has been obtained.)

## Appendix 5: Minor Assent Form

**Project Title:** _____A Multi-modal Intervention to Accelerate Utilization of Human Papilloma Virus Screening for Cervical Cancer in Kiambu County, Kenya _______________________________________________

**Investigator(s):** ____________________

We are doing a research study to help us better understand the utilization of HPV screening amongst women of reproductive age in Kiambu County.

Permission has been granted to undertake this study by ____________________.

If you decide that you want to be part of this study, you will be asked some questions and the information you provide will be recorded in a questionnaire.

There are some things about this study you should know. Some of the questions you will be asked are of an intimate nature and may be embarrassing or make you uncomfortable. If this happens, you may refuse to answer these questions if you so choose. You may also stop the interview at any time. The interview will take approximately half an hour of your time.

If you participate in this study you will help us to learn how to provide effective screening services that can improve health of women.

There are no rewards or any payment to you if you participate.

To protect your privacy, your name will not be recorded on the questionnaire. The questionnaires collected will also be kept confidential and only shared with the study team. When we are finished with this study we will write a report about what was learned. This report will not include your name or that you were in the study.

You do not have to be in this study if you do not want to be. If you decide to stop after we begin, that’s okay too.

If you have questions about the study, you can reach the Principal Investigator, ____________________.

However, if you have questions about your rights as a study participant: You may contact the Ethical Review Committee Secretariat on ____________________

If you decide you want to be in this study, please sign below. Permission will also be sought from your parents to enable you participate in the study.

Signing below is an indication; that the nature of the study has been fully explained to you and you have been given a chance to ask questions and your questions have been answered to your satisfaction; that your participation in this study is entirely voluntary; that you understand that your records will be kept confidential and that you can leave the study at any time.; and that the information provided has enabled you to make a fully informed decision concerning your involvement in the study.

A copy of this consent form will be provided to you.

_____________________________________

Name of Participant

_____________________________________

Signature or Thumbprint

______________________________

Date

**Investigators Statement**

I, the undersigned, have explained to the volunteer in a language she/he understands the procedures to be followed in the study and the risks and benefits involved.

_____________________________________

Name of Interviewer (Research Assistant)

_____________________________________

Signature

______________________________

Date

(This signed form affirms subject eligibility for the study and that informed consent has been obtained.)

## Appendix 6: Questionnaire

**PLERIMINARIES**

P1. County……………………………..

P2. Sub County……………………………..

P3. Ward……………………………..

P4. Research assistant……………………………..

P5. Accompanying CHV……………………………..

P6. Geo-point……………………………..

P6. Respondent Id……………………………..

P7. Date of survey……………………………..

P8. Survey type (Baseline or Endline)……………………………..

1. **Socio-demographic Information**

A1. Age in years……………………………..

A2. Religion (Protestant, Catholic, Muslim or Other)………………………………….

A3. Ethnic group…………………………….

A4. What is your current relationship status?

1. Single
2. Married/Living with partner
3. Separated
4. Divorced
5. Widowed
6. Other (specify) ……………………………..

A5. How long have you been in your current relationship?

(Record in years) …………………………….. N/A……………………………..

A6. Highest completed level of Education

1. No formal education
2. Primary
3. Secondary
4. College/University(Tertiary)

A7. What is your current occupation?

1. Unemployed
2. Employed
3. Self-employed
4. Casual

A8. Who pays for your health expenditure?

1. Employer health insurance
2. Private health insurance
3. Out of pocket (yourself or family member)
4. NHIF
5. **Awareness and Knowledge of cervical cancer and screening**

B1. Before today, have you heard of cervical cancer?

1. No
2. Yes

B2. If you answered ‘Yes’ in B1, what was the source of information on cervical cancer (multiple responses)?

1. Friends or Family
2. Health workers
3. Mainstream media like Radio, TV or Newspapers
4. Social media like Facebook, Twitter or WhatsApp
5. School, Teachers, Lecturers
6. Religious leaders

B3. Before today have you heard of cervical cancer screening?

1. No
2. Yes

B4. If you answered ‘Yes’ in B3, what was the source of information on cervical cancer screening (multiple responses)?

1. Friends or Family
2. Health workers
3. Mainstream media like Radio, TV or Newspapers
4. Social media like Facebook, Twitter or WhatsApp
5. School, Teachers, Lecturers
6. Religious leaders

B5. Do you know a place where cervical cancer screening is done?

1. No
2. Yes

B6. If you answered ‘Yes’ in B5, what type of facility/institution is it?

1. Public hospital

2. Public health centre

3. Faith based facility

4. Private facility

5. NGO based facility

B7. When should someone have their first cervical cancer screening?

1. At marriage

2. When they are sexually active

3. Don’t know

B8. Indicate whether the statements about cervical cancer are **‘True’** or ‘**False’**. If you do not know, indicate **‘Don’t Know’*.***

|  | **True** | **False** | **Don’t Know** |
| --- | --- | --- | --- |
| You are more likely to get cervical cancer if your family member has it. |  |  |  |
| If a woman has an abnormal cervical cancer screening result, she will definitely have cervical cancer. |  |  |  |
| HPV infection increases the risk/probability of developing cervical cancer. |  |  |  |
| Early initiation of sexual intercourse increases the risk of getting cervical cancer. |  |  |  |
| Having multiple sexual partners increases the risk of getting cervical cancer. |  |  |  |
| Having a sexual partner with multiple sexual partners increases the risk of getting cervical cancer? |  |  |  |
| Co-infection with other sexually transmitted infections increases the risk of getting cervical cancer. |  |  |  |
| Having many children/multiparity increases the risk of getting cervical cancer. |  |  |  |
| Immunosuppression due to HIV/AIDS infection increases the risk of getting cervical cancer. |  |  |  |
| Tobacco use increases the risk of getting cervical cancer. |  |  |  |

1. **Attitude towards cervical cancer and screening**

C1. Please indicate whether you strongly agree (SA), agree (A), disagree (D) or strongly disagree (SD) with the statements below;

1. Cervical cancer is a deadly disease ……………………………..
2. Cervical cancer can be prevented ……………………………..
3. Any woman who has ever had sexual intercourse should be screened for cervical cancer ……
4. Screening helps in prevention of cervical cancer ……………………………..
5. Screening for cervical cancer is expensive ……………………………..
6. Cervical cancer screening can be a painful test ……………………………..
7. Cervical cancer screening is embarrassing ……………………………..
8. Cervical cancer screening takes a lot of time ……………………………..
9. **Partner support and barriers in cervical cancer screening**

D1. Do you receive any support from your partner towards cervical cancer screening?

0. No

1. Yes

3. N/A

D2. If you answered ‘Yes’ to D1, what nature of support have you received from your partner towards cervical cancer screening (multiple responses)?

1. Financial support
2. Information
3. Accompanying to health facility
4. Moral/psychological support

D3. If you have ever been screened for cervical cancer or have tried unsuccessfully to get screened, what are the barriers you faced in accessing cervical cancer screening?

1. Lack of information
2. Cost of the tests
3. Accessibility of the facilities
4. Shortage of personnel/HCW
5. Fear of the test and their outcomes
6. Other (specify)………………………………………………………………….
7. **HPV Knowledge,**

E1. Before today, had you ever heard of Human Papillomavirus (HPV)?

1. No
2. Yes

E2. If you answered ‘Yes’ **in E1,** indicate whether the following statement are **‘True’** or **‘False’**. If you do not know, indicate ‘**Don’t Know’.**

1. HPV can cause cervical cancer……………………………….
2. A person could have HPV for many years without knowing it…………………………
3. Having many sexual partners or a partner with many sexual partners increases the risk of getting HPV……………………………….
4. HPV is very rare……………………………….
5. Men **cannot** get HPV……………………………….
6. HPV can be passed on during sexual intercourse……………………………….
7. HPV **always** has visible signs or symptoms……………………………….
8. Using condoms reduces the risk of getting HPV……………………………….
9. HPV can cause HIV/AIDS……………………………….
10. Having sex at an early age increases the risk of getting HPV……………………………
11. HPV can cause genital warts……………………………….
12. HPV can be cured with antibiotics……………………………….
13. **Testing**

F1. Have you ever heard of HPV testing?

1. No
2. Yes
3. Don’t know

F2. If your answer in F1 is ‘Yes’, what was your source of information on HPV testing?

1. Friends or Family
2. Health workers/CHVs
3. Mainstream media like Radio, TV or Newspapers
4. Social media like Facebook, Twitter or WhatsApp
5. School, Teachers, Lecturers
6. Religious leaders

F3. Indicate whether the following statements about HPV testing are **‘True’** or **‘False’**. If you do not know indicate, **‘Don’t know’**

1. If a woman tests positive for HPV she will definitely get cervical cancer…………………
2. An HPV test can tell you how long you have had a HPV Infection……………………….
3. HPV testing is used to indicate/assess if the HPV vaccine is needed……………………..
4. When you have a HPV test you get the results the same day……………………………
5. If a HPV test shows that a woman does not have HPV, her risk of cervical cancer is low……………………………….
6. **HPV Vaccination**

G1. Do have a daughter(s) aged 9-14 years?

1. No
2. Yes

G2. Before today, had you ever heard of HPV vaccination?

1. No
2. Yes

G3. Has your daughter(s) received the HPV vaccine?

1. No
2. Yes
3. Some have
4. **Intent to Test for HPV**

H1. If you had an opportunity to test for HPV today would you do it?

1. No
2. Definitely Yes
3. Maybe
4. **Medical History**

I1. Has a health professional ever told you about your HPV status?

1. No
2. Yes

I2. Have you ever undergone cervical cancer screening?

1. No
2. Yes

I3. If ‘Yes’, what test was done?

1. HPV
2. Pap smear
3. Via Villi
4. Other (specify)

I4. Would you recommend the type of screening you received to other women?

1. No
2. Yes
3. N/A

I5. Give the reasons for recommending or not recommending………………………….

……………………………………………………………………………………………

…………………………………………………………………………………………….

…………………………………………………………………………………………

I6. If you answered ‘Yes’ to I2, how often do you go for cervical cancer screening?

1. Annually

1. Every 3 years
2. Every 5 years
3. Once in a while
4. I can’t remember

I7. If you answered ‘Yes’ to I2, when were you last screened for cervical cancer?

1. Within the last year
2. Within the last 3 years
3. Within the last 5 years
4. I can’t remember

I8. If you answered ‘No’ to I2, why have you never undergone cervical cancer screening?

......................................................................................................................................................

…………………………………………………………………………………………………..

…………………………………………………………………………………………………..

I9. Do you have a sexual partner?

1. No
2. Yes

I10. How often do you use a condom?

1. Never
2. Everytime
3. Sometimes

I11. Have any of your family members ever had cervical cancer?

1. No
2. Yes

I12. Have any of your friends ever had cervical cancer?

1. No
2. Yes

I13. Have you ever had an infection in your reproductive system in the last 5 years?

1. No
2. Yes

I14. If you answered ‘Yes’ to question I13, did you seek treatment?

1. No
2. Yes

## Appendix 7: Summary of Participant Awareness of Cervical Cancer related factors (n=472)

| Awareness Characteristics | n (%) |
| --- | --- |
| Ever heard of Cervical Cancer | Yes – 408 (86.44%) |
| Ever heard of Cervical Cancer Screening | Yes – 335 (70.97%) |
| Know a place for Cervical Cancer Screening | Yes – 297 (62.92%) |
| Ever heard of HPV | Yes – 211 (44.70%) |
| Ever heard of HPV testing | Yes – 129 (27.33%) |
| Ever heard of HPV Vaccination | Yes – 189 (40.04%) |

## Appendix 8: Summary of Participant Knowledge of HPV related factors (n=472)

| Knowledge Characteristics | n (%) |
| --- | --- |
| HPV can cause cervical cancer. | 150 (31.77%) |
| A person could have HPV for many years without knowing it. | 124 (26.27%) |
| Having many sexual partners or a partner with many sexual partners’ increases the risk of getting HPV. | 165 (34.96%) |
| HPV is rare. | 74 (15.68%) |
| Man cannot get HPV. | 70 (14.83%) |
| HPV can be passed on during sexual intercourse. | 148 (31.36%) |
| HPV infection always has visible signs or symptoms. | 6 (1.27%) |
| Using condoms reduces the risk of getting HPV. | 126 (26.69%) |
| HPV can cause HIV/AIDS. | 100 (21.19%) |
| Having sex at an early age increases the risk of getting HPV. | 140 (29.66%) |
| HPV can cause genital warts. | 62 (13.14%) |
| HPV can be cured with antibiotics. | 57 (12.08%) |
| If a woman tests positive for HPV she will definitely get cervical cancer. | 88 (18.64%) |
| A HPV test can tell you how long you have had a HPV infection. | 53 (11.23%) |
| HPV testing is used to indicate/assess if the HPV vaccine is needed. | 40 (8.47%) |
| When you have a HPV test, you get the results the same day. | 64 (13.56%) |
| If a HPV test shows that a woman does not have HPV, her risk of cervical cancer is low. | 96 (20.34%) |

## Appendix 9: Univariate analysis: Characteristics of study participants by screening behaviour (n=340)

| **SOCIO-DEMOGRAPHIC CHARACTERISTICS** | **SCREENED** | **NOT SCREENED** | **OR** | **P VALUE** | **95% CI** |
| --- | --- | --- | --- | --- | --- |
| ***Age (mean, SD)*** | 36.4 (±7.2) | 30.0 (±8.9) | 1.09 | 0.000 | 1.06 – 1.12 |
| ***Religion -*** ***n (%)***  Other (Reference)  Protestant  Catholic | 3 (0.9%) | 6 (1.8%) | **Ref** | **Ref** | **Ref** |
|  | 48 (14.1%) | 187 (55.0%) | 0.73 | 0.635 | 0.20 – 2.70 |
|  | 18 (5.3%) | 78 (22.9%) | 0.58 | 0.434 | 0.15 – 2.28 |
| ***Occupation - n (%)***  Unemployed (Reference)  Employed  Self-employed  Casual |  |  |  |  |  |
|  | 17 (5.0%) | 93 (27.4%) | **Ref** | **Ref** | **Ref** |
|  | 7 (2.1%) | 25 (7.4) | 1.87 | 0.166 | 0.77 – 4.55 |
|  | 37 (10.9%) | 112 (32.9%) | 2.53 | 0.002 | 1.42 – 4.52 |
|  | 8 (2.4%) | 41 (12.1%) | 1.44 | 0.410 | 0.60 – 3.46 |
| ***Level of Education - n (%)***  No formal education  Primary education  Secondary education  Tertiary education |  |  |  |  |  |
|  | 2 (0.6%) | 9 (2.6%) | **Ref** | **Ref** | **Ref** |
|  | 29 (8.5%) | 115 (33.8%) | 1.23 | 0.791 | 0.26 – 5.80 |
|  | 25 (7.4%) | 93 (27.4%) | 1.02 | 0.976 | 0.22 – 4.82 |
|  | 13 (3.8%) | 54 (15.1%) | 1.20 | 0.823 | 0.24 – 5.92 |
| ***Marital Status - n (%)***  Single (never married) (Reference)  Married  Separated / Divorced / Widowed |  |  |  |  |  |
|  | 12 (3.5%) | 47(13.8%) | **Ref** | **Ref** | **Ref** |
|  | 41 (12.1%) | 196 (57.6) | 1.93 | 0.043 | 1.02 – 3.65 |
|  | 16 (4.7%) | 28 (8.2%) | 4.89 | 0.000 | 2.17 – 11.04 |
| ***Sexual Partner - n (%)***  Yes  No  No response |  |  | 1.53 | 0.217 | 0.78 – 2.99 |
|  | 44 (12.9%) | 203 (59.7%) |  |  |  |
|  | 12 (3.5%) | 42 (12.4%) |  |  |  |
|  | 13 (3.8%) | 26 (7.6%) |  |  |  |
| ***Ever had a family member diagnosed with cervical cancer - n (%)***  Yes  No  No response |  |  | 1.61 | 0.216 | 0.76 – 3.41 |
|  | 8 (2.4%) | 30 (8.8%) |  |  |  |
|  | 57 (16.8%) | 233 (68.5%) |  |  |  |
|  | 4 (1.2%) | 8 (2.4%) |  |  |  |
| ***Ever had a friend diagnosed with cervical cancer - n (%)***  Yes  No |  |  | 1.48 | 0.196 | 0.82 – 2.66 |
|  | 18 (5.3%) | 56 (16.5%) |  |  |  |
|  | 51 (15.0%) | 215 (63.2%) |  |  |  |
| ***Source of payment for health services - n (%)***  Employer insurance  Private health insurance  Out of pocket payments  NHIF |  |  |  |  |  |
|  | 2 (0.6%) | 2 (0.6%) | **Ref** | **Ref** | **Ref** |
|  | 2 (0.6%) | 4 (1.2%) | 0.5 | 0.600 | 0.04 – 6.68 |
|  | 43 (12.7%) | 191 (56.2%) | 0.21 | 0.128 | 0.03 – 1.56 |
|  | 22 (6.5%) | 74 (21.8%) | 0.30 | 0.238 | 0.04 – 2.23 |

| **PARTICIPANT AWARENESS OF CERVICAL CANCER RELATED FACTORS, *n (%)*** | **SCREENED** | **NOT SCREENED** | **OR** | **P VALUE** | **95% CI** |
| --- | --- | --- | --- | --- | --- |
| ***Ever heard of cervical cancer***  Yes  No |  |  | 6.76 | 0.009 | 1.62 – 28.25 |
|  | 68 (20.0%) | 241 (70.9%) |  |  |  |
|  | 1 (0.3%) | 30 (8.8%) |  |  |  |
| ***Ever heard of cervical cancer screening***  Yes  No |  |  | 26.24 | 0.001 | 3.60 – 191.48 |
|  | 60 (17.6%) | 204 (60.0%) |  |  |  |
|  | 1 (0.3%) | 56 (16.5%) |  |  |  |
| ***Know a place for cervical cancer screening***  Yes  No |  |  | 12.65 | 0.000 | 3.90 – 41.05 |
|  | 58 (17.1%) | 179 (52.6%) |  |  |  |
|  | 11 (3.3%) | 92 (27.0%) |  |  |  |
| ***Ever heard of HPV***  Yes  No |  |  | 4.25 | 0.000 | 2.45 – 7.36 |
|  | 51 (15.0%) | 117 (34.4%) |  |  |  |
|  | 18 (5.3%) | 154 (45.3%) |  |  |  |
| ***Ever heard of HPV testing***  Yes  No |  |  | 4.86 | 0.000 | 2.90 – 8.14 |
|  | 39 (11.5%) | 70 (20.6%) |  |  |  |
|  | 30 (8.8%) | 201 (59.1%) |  |  |  |
| ***Ever heard of HPV vaccination***  Yes  No |  |  | 5.40 | 0.000 | 3.11 – 9.38 |
|  | 51 (15.0%) | 104 (30.6%) |  |  |  |
|  | 18 (5.3%) | 167 (49.1%) |  |  |  |

| **KNOWLEDGE, mean (SD)** | **SCREENED** | **NOT SCREENED** | **OR** | **P VALUE** | **95% CI** |
| --- | --- | --- | --- | --- | --- |
| Knowledge of cervical cancer related factors | 6.4 (±2.4) | 4.2 (±3.0) | 1.33 | 0.000 | 1.20 – 1.47 |
| Knowledge of HPV related factors | 5.9 (±4.4) | 2.8 (±3.9) | 1.18 | 0.000 | 1.12 – 1.25 |
|  |  |  |  |  |  |
| **ATTITUDE, mean (SD)** | **SCREENED** | **NOT SCREENED** | **OR** | **P VALUE** | **95% CI** |
| Attitude towards cervical cancer related factors | 5.2 (±1.5) | 4.0 (±1.5) | 1.81 | 0.000 | 1.50 – 2.18 |
|  |  |  |  |  |  |
| **PARTNER SUPPORT, n (%)** | **SCREENED** | **NOT SCREENED** | **OR** | **P VALUE** | **95% CI** |
| Yes  No  N/A | 31 (9.1%) | 22 (6.5%) | 1.05 | 0.721 | 0.80 – 1.37 |
|  | 22 (6.5%) | 169 (49.7%) |  |  |  |
|  | 16 (4.7%) | 80 (23.5%) |  |  |  |

## Appendix 10: Nature of Partner Support provided to women by their partners (n=59)


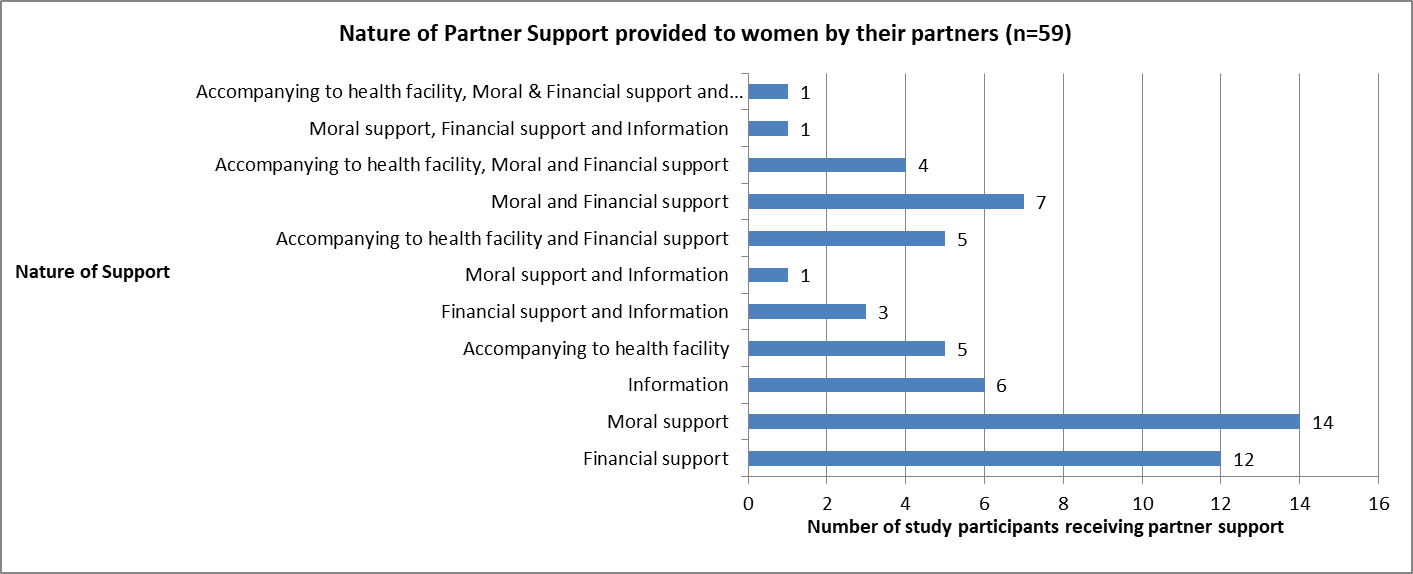


## Appendix 11: Participant Barriers to Accessing Health Services (n=472)

|  | Barriers to Accessing Health Services | n (%) |
| --- | --- | --- |
| 1. | Lack of information | 64 (13.56%) |
| 2. | Costs of tests | 7 (1.48%) |
| 3. | Accessibility of the facilities | 6 (1.27%) |
| 4. | Shortage of personnel / healthcare workers | 2 (0.42%) |
| 5. | **Fear of tests and outcomes** | **90 (19.06%)** |
| 6. | Other | 143 (30.30%) |
| 7. | Lack of information & Costs of tests | 4 (0.85%) |
| 8. | Lack of information & Accessibility of the facilities | 1 (0.21%) |
| 9. | Lack of information & Fear of tests and outcomes | 59 (12.5%) |
| 10. | Lack of information & Other | 2 (0.42%) |
| 11. | Costs of tests & Accessibility of the facilities | 1 (0.21%) |
| 12. | Costs of tests & Fear of tests and outcomes | 9 (1.91%) |
| 13. | Costs of tests & Other | 1 (0.21%) |
| 14. | Accessibility of the facilities & Fear of tests and outcomes | 5 (1.06%) |
| 15. | Shortage of personnel / healthcare workers & Fear of tests and outcomes | 1 (0.21%) |
| 16. | Fear of tests and outcomes & Other | 7 (1.48%) |
| 17. | Lack of information, Costs of tests & Accessibility of the facilities | 1 (0.21%) |
| 18. | Lack of information, Costs of tests & Fear of tests and outcomes | 12 (2.54%) |
| 19. | Lack of information, Accessibility of the facilities & Fear of tests and outcomes | 1 (0.21%) |
| 20. | Lack of information, Fear of tests and outcomes & Other | 1 (0.21%) |
| 21. | Costs of tests, Accessibility of the facilities & Fear of tests and outcomes | 1 (0.21%) |
| 22. | Lack of information, Costs of tests, Accessibility of the facilities & Fear of tests and outcomes | 29 (6.14%) |
| 23. | Lack of information, Costs of tests, Accessibility of the facilities, Shortage of personnel / healthcare workers & Fear of tests and outcomes | 22 (4.66%) |
| 24. | Lack of information, Costs of tests, Accessibility of the facilities, Fear of tests and outcomes & Other | 1 (0.21%) |
| 25. | Lack of information, Costs of tests, Accessibility of the facilities, Shortage of personnel / healthcare workers, Fear of tests and outcomes & Other | 2 (0.42%) |
